# West Nile Virus–associated Flaccid Paralysis Outcome

**DOI:** 10.3201/eid1203.050643

**Published:** 2006-03

**Authors:** James J. Sejvar, Amy V. Bode, Anthony A. Marfin, Grant L. Campbell, John Pape, Brad J. Biggerstaff, Lyle R. Petersen

**Affiliations:** * Centers for Disease Control and Prevention (CDC), Atlanta, Georgia, USA;; † CDC, Fort Collins, Colorado, USA;; ‡ Colorado Department of Health and Environment, Denver, Colorado, USA

**Keywords:** West Nile virus, paralysis, poliomyelitis, outcome, dispatch

## Abstract

We report 1-year follow-up data from a longitudinal prospective cohort study of patients with West Nile virus–associated paralysis. As in the 4-month follow-up, a variety of recovery patterns were observed, but persistent weakness was frequent. Respiratory involvement was associated with considerable illness and death.

During the summer and fall of 2003, we conducted a prospective, population-based study among residents of Larimer, Weld, and Boulder Counties in northern Colorado in whom West Nile virus (WNV)–associated paralysis developed (*1*). We identified 32 patients with paralysis and acute WNV infection. Clinical or neurodiagnostic findings suggested a poliomyelitis-like syndrome in 27 (84%) and a Guillain-Barré–like syndrome (GBLS) in 4 (13%); 1 patient had brachial plexus involvement alone. The cohort was reevaluated 4 months later, at which time 3 patients with respiratory failure had died, 2 remained intubated, 25 showed varying degrees of improvement, and 2 were lost to follow-up. Here, we describe the results of a 1-year follow-up evaluation of the 27 remaining cohort members and describe the patterns of recovery, persistence of symptoms and signs, and long-term outcome.

## The Study

**Figure A1:**
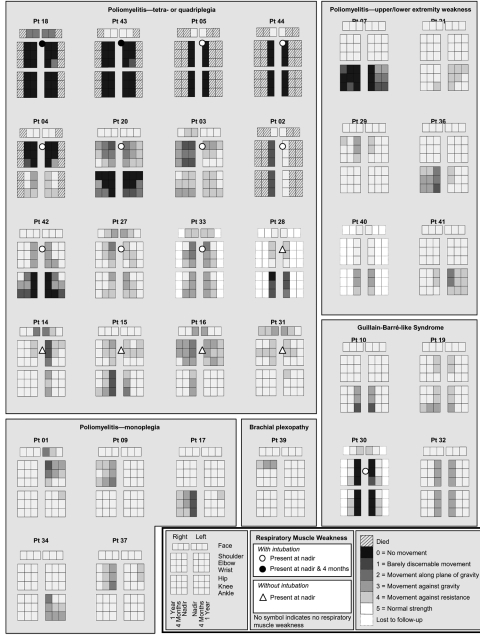
Patterns of weakness at strength nadir, 4 months, and 1 year in patients with acute paralysis and West Nile virus infection. Shadings indicate strength scoring on manual muscle testing and represent the average strength score of all tested muscles proximally (upper extremity: shoulder adduction/abduction, arm internal/external rotation; lower extremity: hip flexion/extension, thigh adduction/abduction), medially (upper extremity: elbow flexion/extension, pronation/supination; lower extremity: knee flexion/extension), and distally (upper extremity: wrist flexion/extension, finger flexion/extension; lower extremity: ankle plantarflexion/dorsiflexion, foot inversion/eversion, toe flexion/extension) in each limb and the facial muscles; scores were rounded down to the lowest whole number. Patients with respiratory weakness are indicated by circles (intubated) or triangles (not intubated). All patients surviving to 1 year were successfully extubated.

By 1 year, 3 of the remaining cohort members had died (all with respiratory involvement), and an additional 2 persons (1 with poliomyelitis-like syndrome, 1 with GBLS) were lost to follow-up, leaving 22 patients in the 1-year cohort. Various degrees of strength improvement by manual muscle testing (MMT) using the Medical Research Council (MRC) 1–5 scale (*2*) were seen in the 18 of 27 patients with poliomyelitis-like syndrome at 1 year (Figure A1). Greater gains in MMT scores were seen between strength nadir and 4 months than between 4 months and 1 year by both subjective patient description and by serial MMT. Using a proportional odds model (SUDAAN, version 9.0.1, Research Triangle Institute, Research Triangle, NC, USA), we found that MMT scores improved over the 3 evaluations (p<0.001, adjusting for a significant MMT site effect), with the odds ratio for 4 months (relative to the nadir) of 0.40 (95% confidence interval [CI] 0.25–0.63) and for 1 year of 0.23 (95% CI 0.14–0.36), supporting subjective and objective assessment. Facial weakness, which had been present in 10 patients, had completely resolved in all patients by the 1-year follow-up.

One person (patient 27, Figure A1) who initially had quadriplegia and respiratory involvement had returned to pre-illness strength and had no detectable weakness on MMT at 1-year follow-up. Four persons (3 with tetra- or quadriplegia [patients 14, 15, and 31] and 1 with monoplegia [patient 37]) had achieved near-baseline strength (trace weakness on MMT) in affected limbs and reported little or no functional difficulty. Between the 4-month and 1-year follow-up, 4 persons (patients 3, 9, 16, 21) experienced measurable continued improvement between 4 months and 1 year (improvement in MMT score in at least 1 affected limb by >1 scale points), and 9 persons (patients 1, 7, 17, 20, 29, 34, 36, 41, 42) experienced little or no subsequent improvement. Four patients (7, 17, 29, 37) had a 1-point strength decrease in certain muscle groups and specific ranges of motion on MMT between 4 months and 1 year; however, overall strength for major joint ranges of motion or the entire limb did not decrease. All patients employed before illness onset (n = 11) had returned to work, 9 full-time and 2 part-time. Two persons were still using ambulatory aids (canes); 2 others continued to use leg braces.

Persistent symptoms and neurologic signs were observed in 10 persons (Table). Dependent edema and skin changes in affected limbs not present at 4 months were observed in 6 patients. In 1 patient, a deep venous thrombosis of an affected monoplegic leg had developed, presumably because of lack of leg movement. Fatigue was the most commonly reported subjective symptom (11, 56%), followed by myalgias/body pains (6, 27%), anxiety and depression (5, 23%), and mental/cognitive complaints and muscle cramps (4, 18% each).

**Table:** Signs and symptoms in 32 patients with West Nile virus (WNV)–associated paralysis

Sign/symptom	Acute infection, N = 32, no. (%)	4-mo followup, N = 27, no. (%)	1-y followup, N = 22, no. (%)
Fever (temperature >38°C)	29 (91)	0	0
Nausea (with or without vomiting)	26 (81)	0	0
Headache	28 (88)	5 (19)	3 (11)
Altered mental status	16 (50)	0	1 (5)
Meningismus	10 (31)	0	0
Rash	4 (13)	0	0
WNV-associated neurologic features*			
Tremor	21 (66)	8 (25)	9* (41)
Myoclonus	15 (47)	2 (6)	3* (14)
Parkinsonism	8 (25)	2 (6)	5* (23)
Cerebellar ataxia	3 (9)	2 (6)	1 (5)5
Limb atrophy	0	17 (53)	10 (45)

*An apparent increase in the number of persons with tremor, myoclonus, and parkinsonism between 4 mo and 1 y is reflective of detection of these movement disorders in persons who were initially flaccid/immobile, nonambulatory, or too functionally impaired to assess.

One person with GBLS was lost to follow-up. Of the remaining 3 patients, 2 (patients 10, 32) had returned to baseline in terms of strength and overall functioning (Figure A1). The patient with brachial plexopathy and associated isolated shoulder abduction weakness had no additional strength gains in affected muscle groups at 1 year but continued to be unhampered functionally by this deficit.

By the 1-year follow-up, an additional 3 patients with respiratory failure had died (at 4.5 months, 5 months, and 7 months, respectively, after symptom onset). Of the 6 deaths, cause of death was withdrawal of ventilatory support in 5 and cardiac arrest in 1. No persons intubated for >4 months survived. All 6 surviving patients had been discharged from care facilities and were living at home. One patient with GBLS and 1 with poliomyelitis-like syndrome were lost to follow-up by 1 year. Four of the 6 survivors required repeated episodes of extubation and reintubation before being permanently extubated. Of the 4 patients evaluated at 1 year, 2 still required assistance with all daily activities and required supplemental oxygen. The other 2 patients experienced substantial improvements in strength and functional ability, living independently and working at 1 year; however, both continued to experience persistent parkinsonism, tremor, and fatigue. All patients with respiratory involvement continued to experience orthopnea, dyspnea on exertion, and weakness of cough.

Of the 5 patients who experienced shortness of breath and had diagnostic evidence of respiratory muscle dysfunction but did not require intubation, 2 required supplemental oxygen at some point during their recovery. At 1 year, 3 patients continued to experience substantial orthopnea and dyspnea on exertion, and 2 had no respiratory symptoms.

## Conclusions

The 1-year follow-up findings among this cohort of persons with WNV-associated paralysis demonstrate a spectrum of functional and strength outcomes. Among survivors, all demonstrated at least some improvement in strength of affected muscles, but improvement varied substantially. Consistent with findings at 4 months, those persons with less profound initial weakness demonstrated the greatest strength gains at 1 year. Strength gains among patients were more substantial between the nadir and 4 months than in the following months, in which a plateau was reached and less improvement was noted. The MRC scale of MMT is not linear, and this slowing of improvement may be attributable to the inherent nature of the MMT scoring system. However, this pattern of improvement is consistent with observations of recovery from poliovirus infection, in which most strength gains occur within 6 months (*3*). Persons with GBLS experienced complete or near complete recovery, a commonly reported outcome of this syndrome (*4*). Facial weakness was associated with favorable prognosis; however, resolution of weakness took >5 months in some cases.

Respiratory involvement was associated with considerable death and illness; the death rate was 50%. With the exception of 1 patient, death was due to a voluntary withdrawal of ventilatory support. In 2 of these patients, cognition and awareness were intact at the time of support withdrawal. Successful extubation among surviving patients with respiratory involvement occurred only after prolonged weaning periods (mean duration of intubation 66 days) (*1*) and often, multiple episodes of extubation and reintubation. At the 1-year follow-up, 2 of the surviving patients with respiratory involvement had severe disability and required assistance with all activities. However, 2 others with initial respiratory involvement did well; 1 experienced complete strength recovery and the other experienced recovery to the point of functional independence. Predictors of favorable outcome are unknown, but the 2 persons with favorable outcome were relatively young (45 and 43 years of age, respectively) and had no preexisting medical conditions. In contrast, all but 1 person who died or had a poor outcome were >56 years of age, and 4 had noteworthy prior medical conditions. Only 1 of the 5 patients with respiratory paralysis and quadriplegia from poliomyelitis-like syndrome survived, but that patient made a full and complete recovery; thus, respiratory paralysis and quadriplegia do not appear to universally indicate poor outcome. All persons recovering from respiratory failure continued to describe mild persistent orthopnea and exertional dyspnea. Respiratory paralysis appears to be a long-term manifestation of WNV poliomyelitis-like syndrome associated with substantial morbidity.

Although small decreases in strength of specific movements at specific joints were noted in a few persons between 4 months and 1 year (Figure A1), these decreases were minor and observed in isolated ranges of motion, and most likely reflected a combination of MMT measurement variability, patient effort, and the method used to calculate strength scores. Only 1 person displayed worsening in strength of a major joint or limb after initial improvement; a decrement in strength from the 4-month time point was noted in the left upper arm of patient 15 because of limitation of testing of this limb from shoulder arthritis pain. A relapse or subsequent worsening in strength after an initial period of improvement was not observed in these patients, although such phenomena have been observed in patients with poliovirus infection (*5*). The possibility of a "postpoliomyelitis syndrome" (*6*) (e.g., development of weakness in a previously affected limb years after recovery) is unknown and will require longer term assessment.

Persistent associated neurologic signs and symptoms were seen or reported in nearly all patients (Table). Atrophy of affected limbs was common. Dependent edema, presumably due to compartmental muscle weakness and inability to augment blood circulation by muscular contraction, had been present to a mild degree in 2 persons at 4-month follow-up but was seen in 6 persons at 1 year. The development of a deep venous thrombosis in 1 person with leg paresis reinforces the need for vigilance for this complication in persons with severe weakness and lack of mobility.

In summary, the longer-term outcome of WNV poliomyelitis-like syndrome appears to be more heterogeneous than preliminary data may have suggested, with some persons experiencing little neurologic and functional improvement and others experiencing substantial gains. The degree of initial weakness appears to be an indicator of subsequent long-term outcome. WNV poliomyelitis-like syndrome with respiratory involvement is a condition associated with considerable death and illness; however, substantial and even dramatic recovery is sometimes observed in such patients. Persistent quadriplegia and respiratory failure lasting >4 months may indicate poor prognosis. Continued long-term assessment of patients recovering from WNV-associated paralysis is needed to fully discern the spectrum of possible outcomes.
